# Supplementary material for: NAD-dependent dehydrogenases enable efficient growth of Paracoccus denitrificans on the PET monomer ethylene glycol
Source: Nat Commun. 2025 Jul 1;16:5845. doi: 10.1038/s41467-025-61056-x (PMC12214560; doi:10.1038/s41467-025-61056-x)
Supplement: Supplementary file 1 — Supplementary Information [file 41467_2025_61056_MOESM1_ESM.pdf]

NAD-dependent dehydrogenases enable efficient growth of *Paracoccus denitrificans* on the PET monomer ethylene glycol

Ren *et al.*

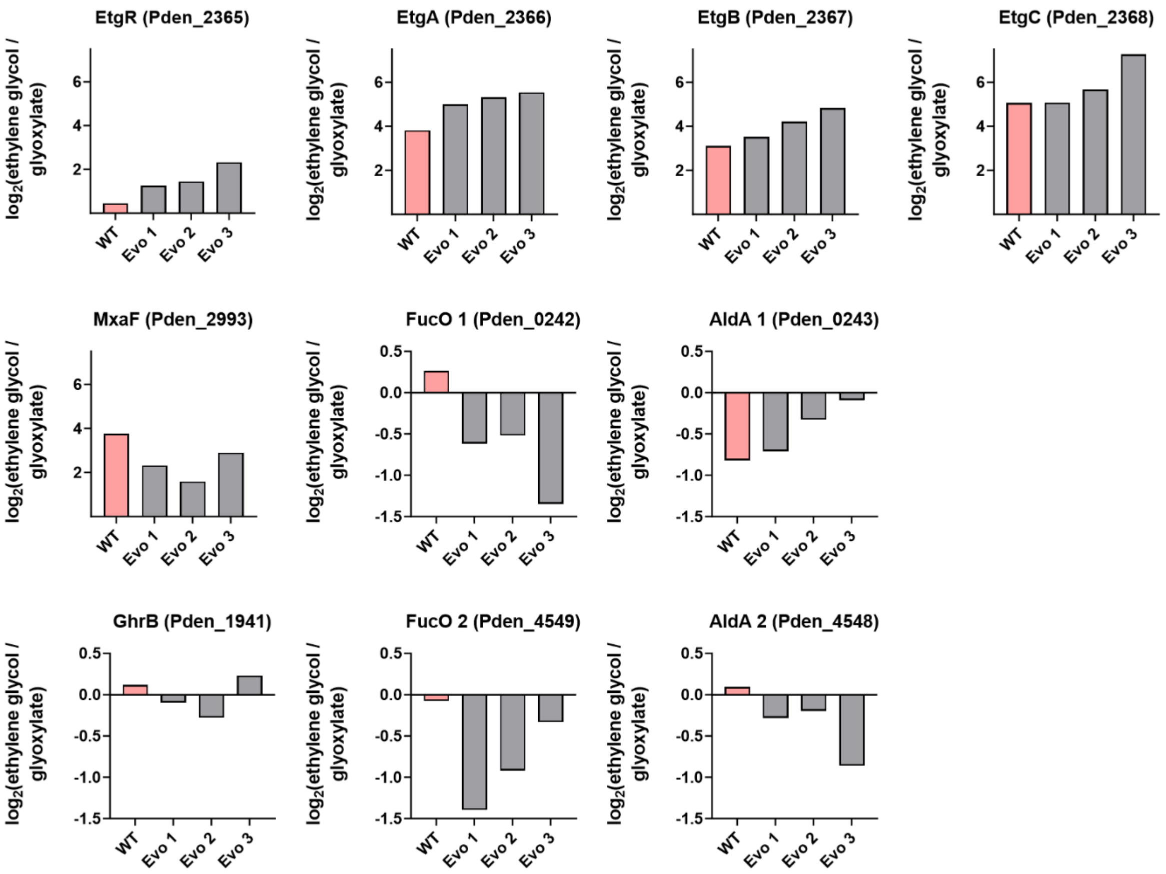

Supplementary Figure 1. Proteome analysis demonstrates upregulation of the *etg* gene cluster during growth on ethylene glycol. The $\log_2$ fold change of EtgR/A/B/C in *P. denitrificans* WT and three evolved isolates growing on ethylene glycol compared to *P. denitrificans* WT growing on glyoxylate is shown. Similarly, the upregulation of MxaF is shown. In contrast, other alcohol and aldehyde dehydrogenases (FucO1/2, AldA1/2) as well as glyoxylate reductase (GhrB) either remained nearly unchanged or were downregulated. Source data are provided as a Source Data file.

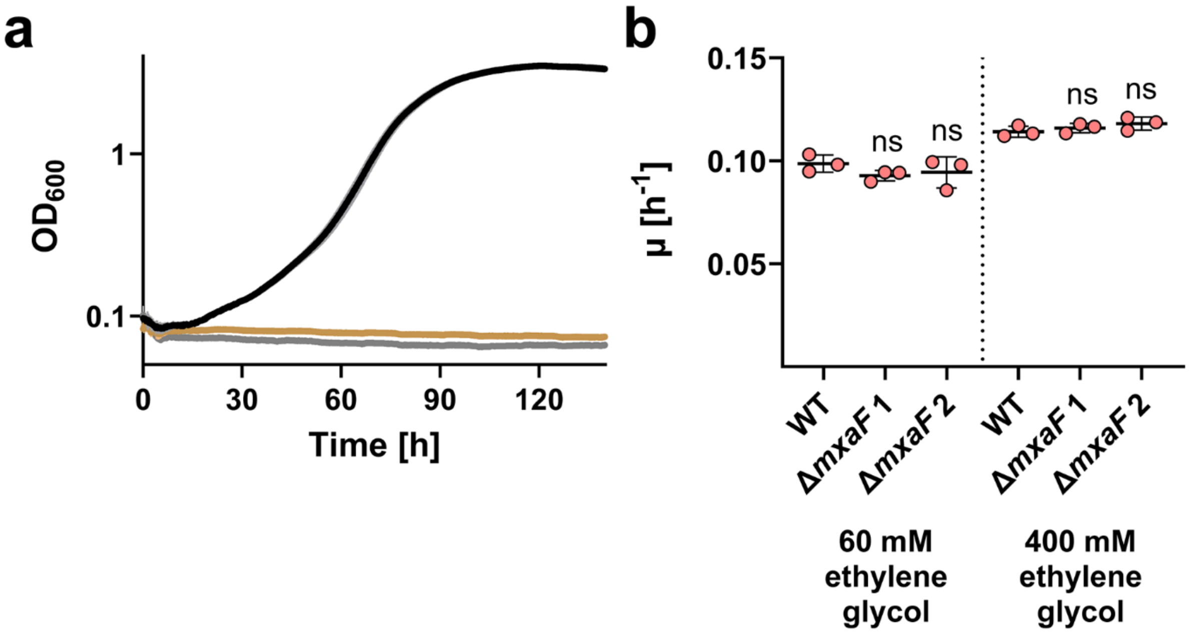

Supplementary Figure 2. Growth of *P. denitrificans* on methanol. **a**, Growth curves of *P. denitrificans* on 120 mM methanol, measured for the wild-type strain (black), and the gene deletion strains Δ*mxaF* 1 and 2 (grey and light brown). Data are the mean ± s.d. of *n* = 6 independently grown cultures, with error bars shown in lighter colors. **b**, Growth rates of *P. denitrificans* WT and Δ*mxaF* 1 and 2 on 60 and 400 mM ethylene glycol. Data are the mean ± s.d. of *n* = 3 independently grown cultures. Results were compared using an unpaired *t*-test with Welch's correction in GraphPad Prism 8.1.1. ns: not significantly different from WT (*p* values for 60 mM ethylene glycol: 0.1215, 0.4522; 400 mM ethylene glycol: 0.4259, 0.1776). Source data are provided as a Source Data file.

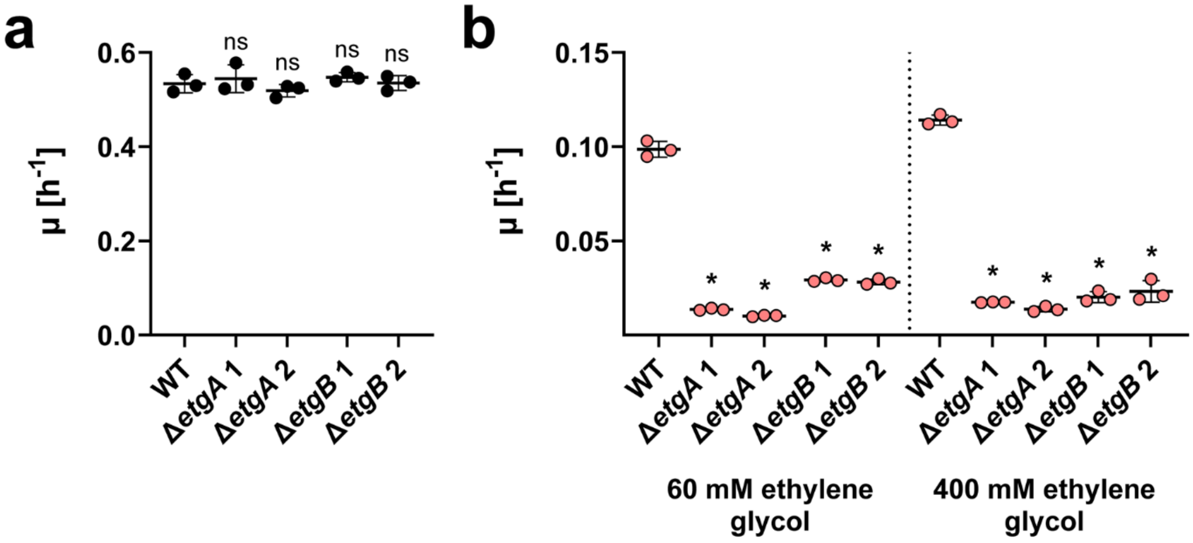

Supplementary Figure 3. Growth of *P. denitrificans* Δ*etgA* and Δ*etgB*. **a**, Growth rates of *P. denitrificans* WT and the Δ*etgA* 1/2 and Δ*etgB* 1/2 gene deletion strains on 30 mM succinate. ns: not significantly different from WT (*p* values: 0.6306, 0.3452, 0.3430, 0.9154). **b**, Growth rates of *P. denitrificans* WT and the Δ*etgA* 1/2 and Δ*etgB* 1/2 gene deletion strains on 60 and 400 mM ethylene glycol. *: significantly different from WT (*p* values for 60 mM ethylene glycol: 0.0006, 0.0007, 0.0007, 0.0004; 400 mM ethylene glycol: 0.0002, 0.0001, 0.0001, 0.0002). For **a** and **b**, data are the mean ± s.d. of *n* = 3 independently grown cultures. Results were compared using an unpaired *t*-test with Welch's correction in GraphPad Prism 8.1.1. Source data are provided as a Source Data file.

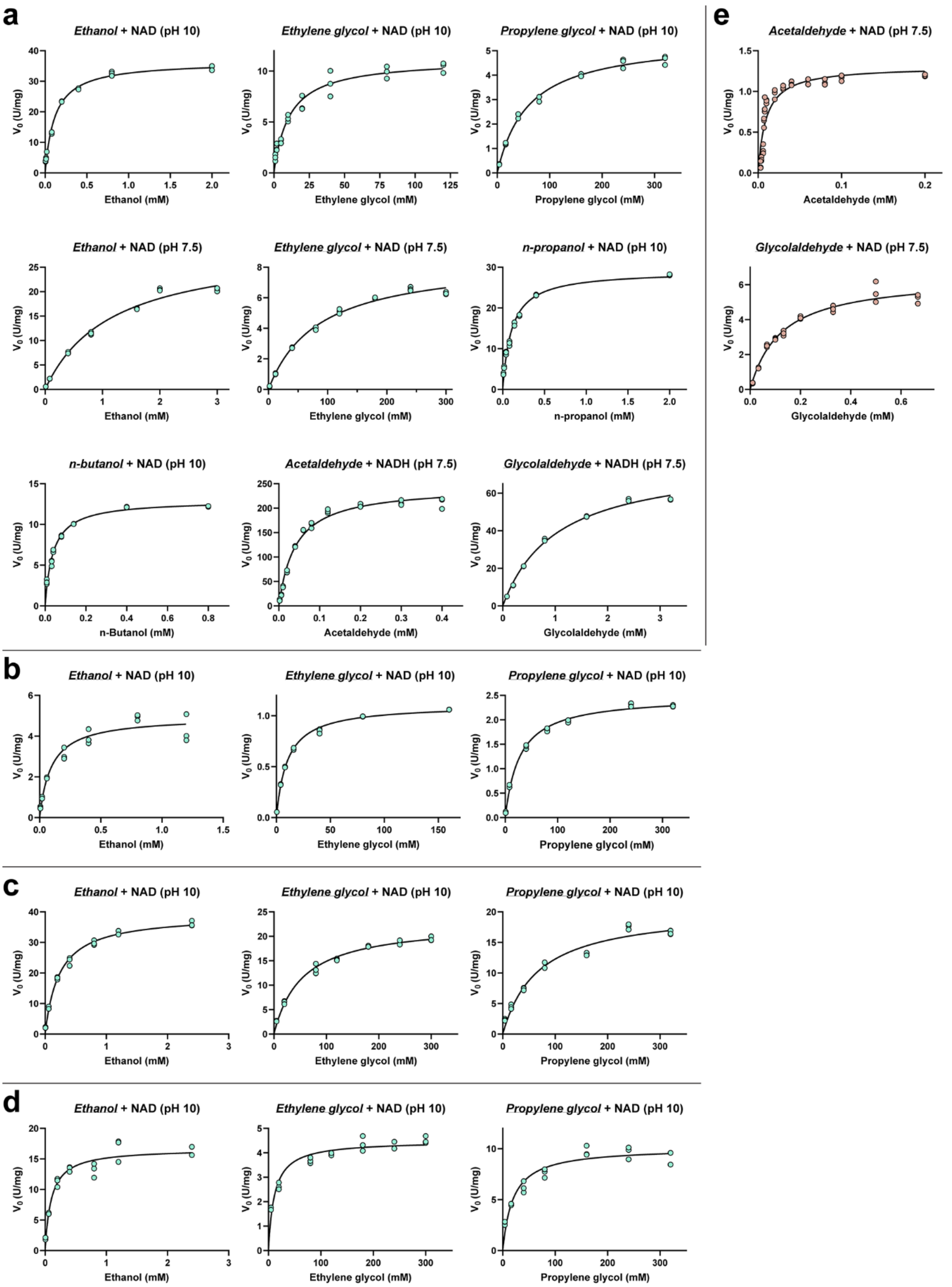

Supplementary Figure 4. Michaelis–Menten kinetics of all enzyme reactions characterized in this study. **a**, Michaelis–Menten kinetics for EtgB. **b**, Michaelis–Menten kinetics for EtgB T44S. **c**, Michaelis–Menten kinetics for EtgB H47N. **d**, Michaelis–Menten kinetics for EtgB T44S H47N. **e**, Michaelis–Menten kinetics for EtgA. **a**–**e**, Data are shown from n = 3 independent experiments at different substrate concentrations. The data are summarized in Table 1. Source data are provided as a Source Data file.

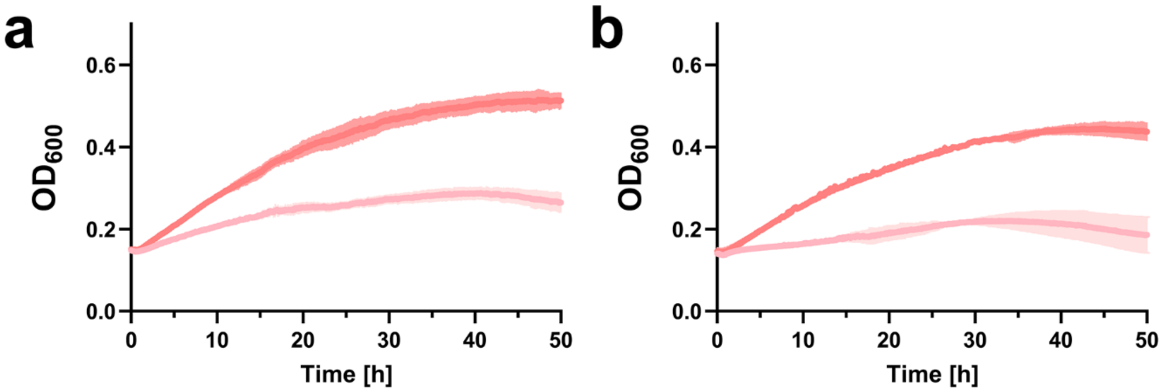

Supplementary Figure 5. Growth of *E. coli* on ethylene glycol. Growth curves of *E. coli* K-12 WT (light pink) and *E. coli* K-12 pZ-ASS-EtgB (light red) on 60 (**a**) and 200 mM (**b**) ethylene glycol. Data are the mean ± s.d. of $n = 3$ independently grown cultures, with error bars shown in lighter colors. Source data are provided as a Source Data file.

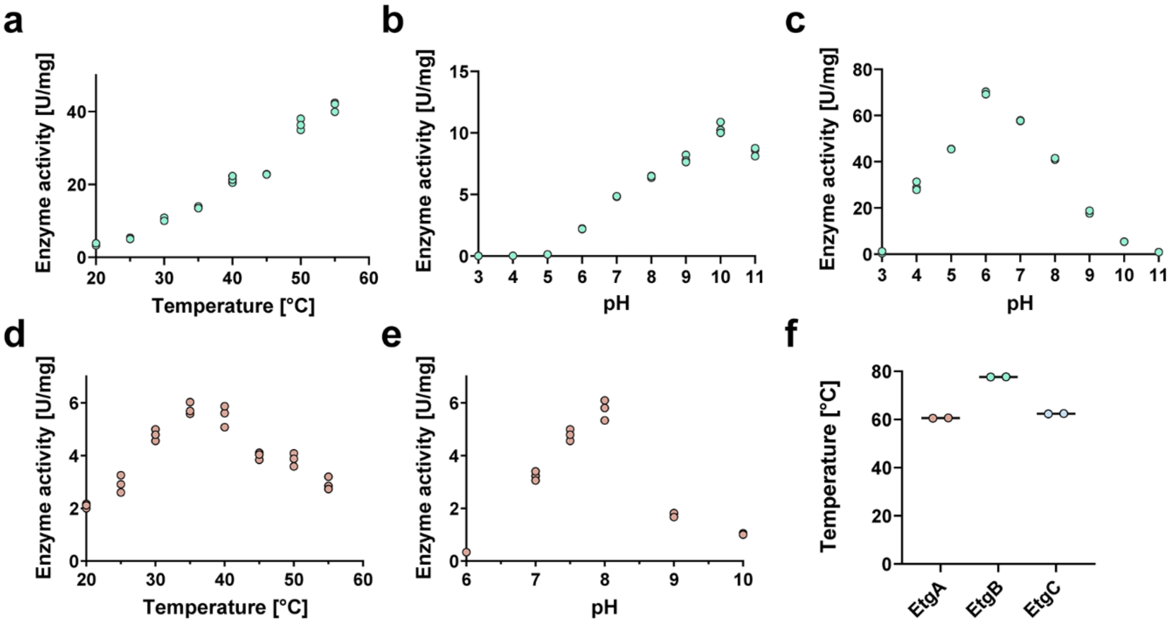

Supplementary Figure 6. Enzyme activities of EtgB and EtgA are dependent on temperature and pH. a, Oxidation of 200 mM ethylene glycol by EtgB with 2.4 mM NAD^+ as cofactor was measured at temperatures between 20 °C and 55 °C. **b**, Oxidation of 200 mM ethylene glycol by EtgB with 2.4 mM NAD^+ as cofactor was measured at pH values from 3 to 11. **c**, Reduction of 2.4 mM glycolaldehyde by EtgB with 0.2 mM NADH as cofactor was measured at pH values from 3 to 11. **d**, Oxidation of 0.6 mM glycolaldehyde by EtgA with 2.4 mM NAD^+ as cofactor was measured at temperatures between 20 °C and 55 °C. **e**, Oxidation of 0.6 mM glycolaldehyde by EtgA with 2.4 mM NAD^+ as cofactor was measured at pH values from 6 to 10. **f**, Unfolding temperatures of EtgA, EtgB, and EtgC. For **a** to **e**, the results of $n = 3$ independent experiments are shown; for **f**, the results of $n = 2$ independent experiments are shown. Source data are provided as a Source Data file.

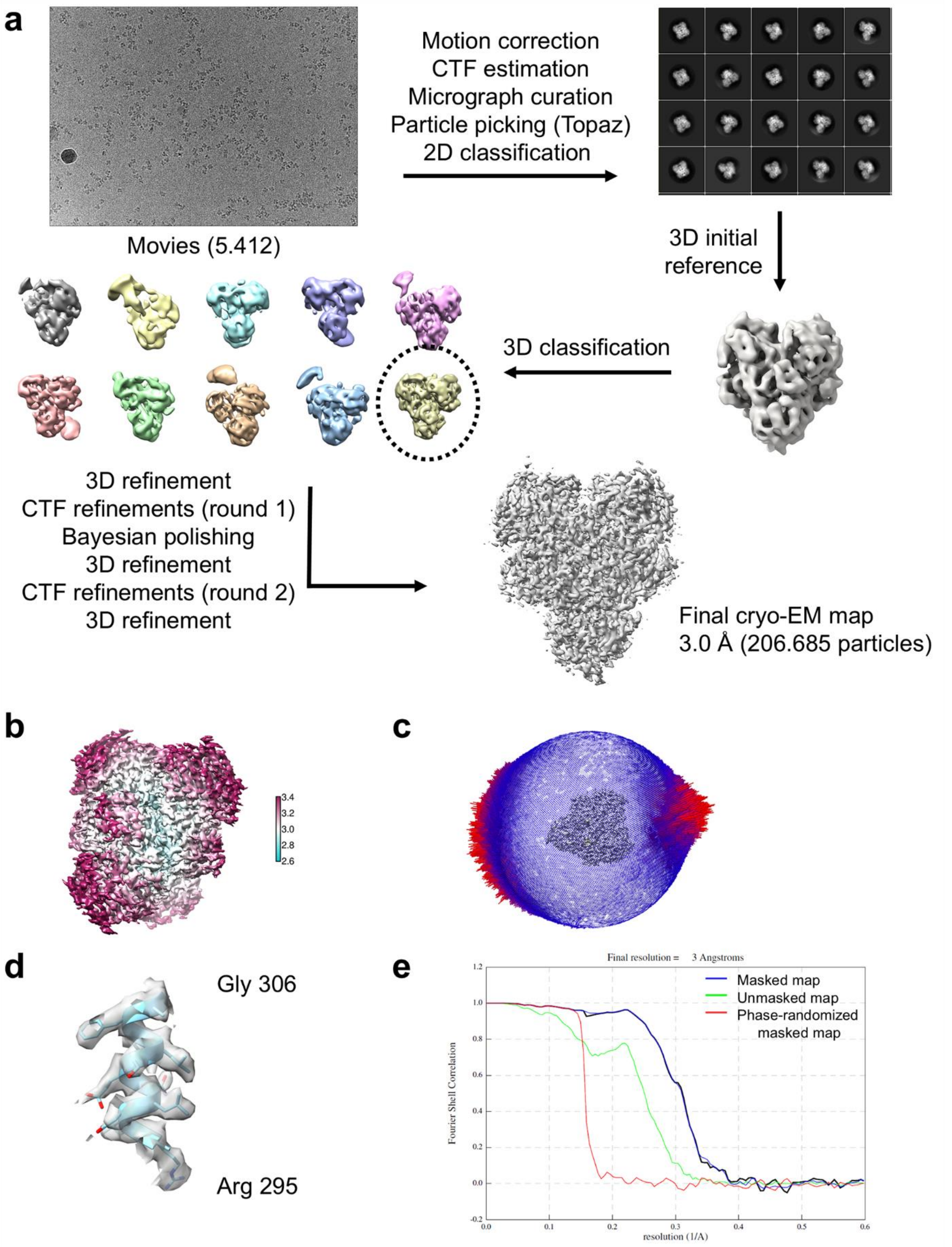

Supplementary Figure 7. **Cryo-EM data processing and evaluation for EtgB. a**, Processing pipeline of EtgA with representative micrograph, 2D classes, 3D classes and final 3D model. **b**, Local resolution map ranging from 2.8 Å in blue to 3.4 Å in red. **c**, Orientational distribution map. **d**, Detail of the cryo-EM map showing a helix with atomic model shown in sticks and the cryo-EM map in transparent surface. **e**, FSC curves for masked and unmasked maps.

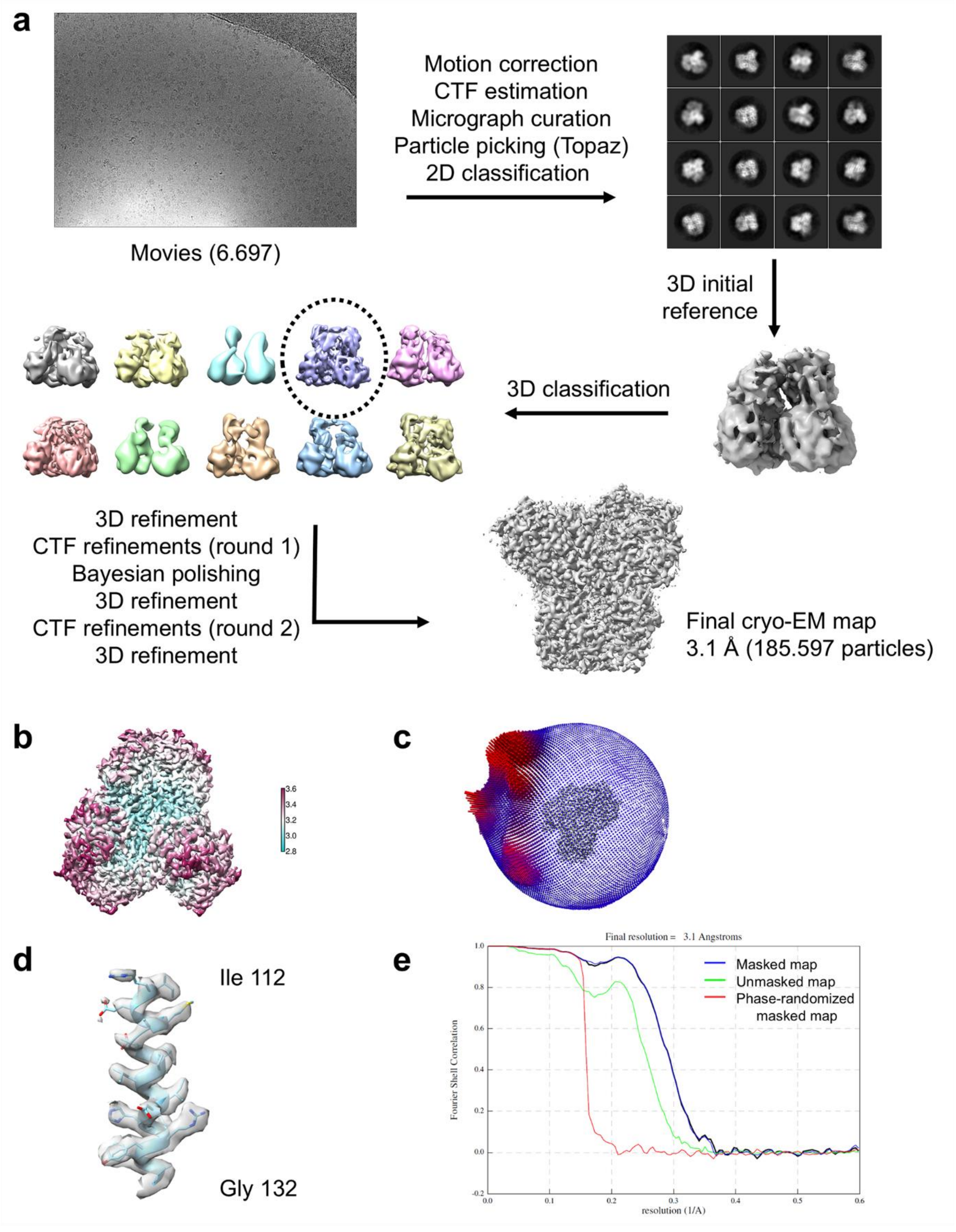

Supplementary Figure 8. **Cryo-EM data processing and evaluation for EtgA. a**, Processing pipeline of EtgA with representative micrograph, 2D classes, 3D classes and final 3D model. **b**, Local resolution map ranging from 2.8 Å in blue to 3.6 Å in red. **c**, Orientational distribution map. **d**, Detail of the cryo-EM map showing a helix with atomic model shown in sticks and the cryo-EM map in transparent surface. **e**, FSC curves for masked and unmasked maps.

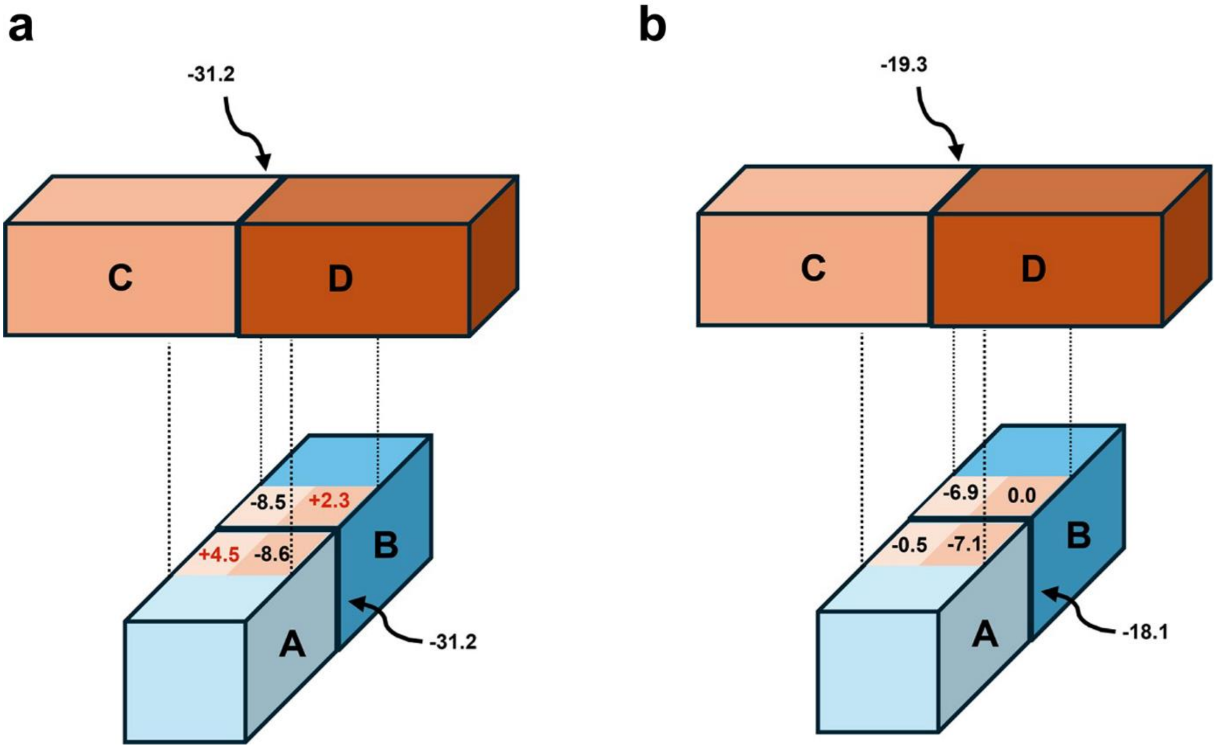

Supplementary Figure 9. Comparison of the tetramer interfaces in EtgA and EtgB. a, Schematic representation of the EtgA 'dimer-of-dimers', showing the A:B dimer in light blue and blue, and the C:D dimer in orange and brown. The inter-dimer interfaces are shown as light-orange and light-brown areas on the A:B dimer. Gibbs free energy (ΔG) values are shown in kcal/mol for each of the interfaces. The main dimer interfaces, A:B and C:D, show DG values of -31.2 kcal/mol, indicative of positive protein affinity. In contrast, the inter-dimer contacts between B:D and A:C show unfavorable ΔG values of +2.3 and +4.5 kcal/mol, respectively (shown in red font). **b**, Similar analysis for EtgB, where the unfavorable ΔG values are not observed.

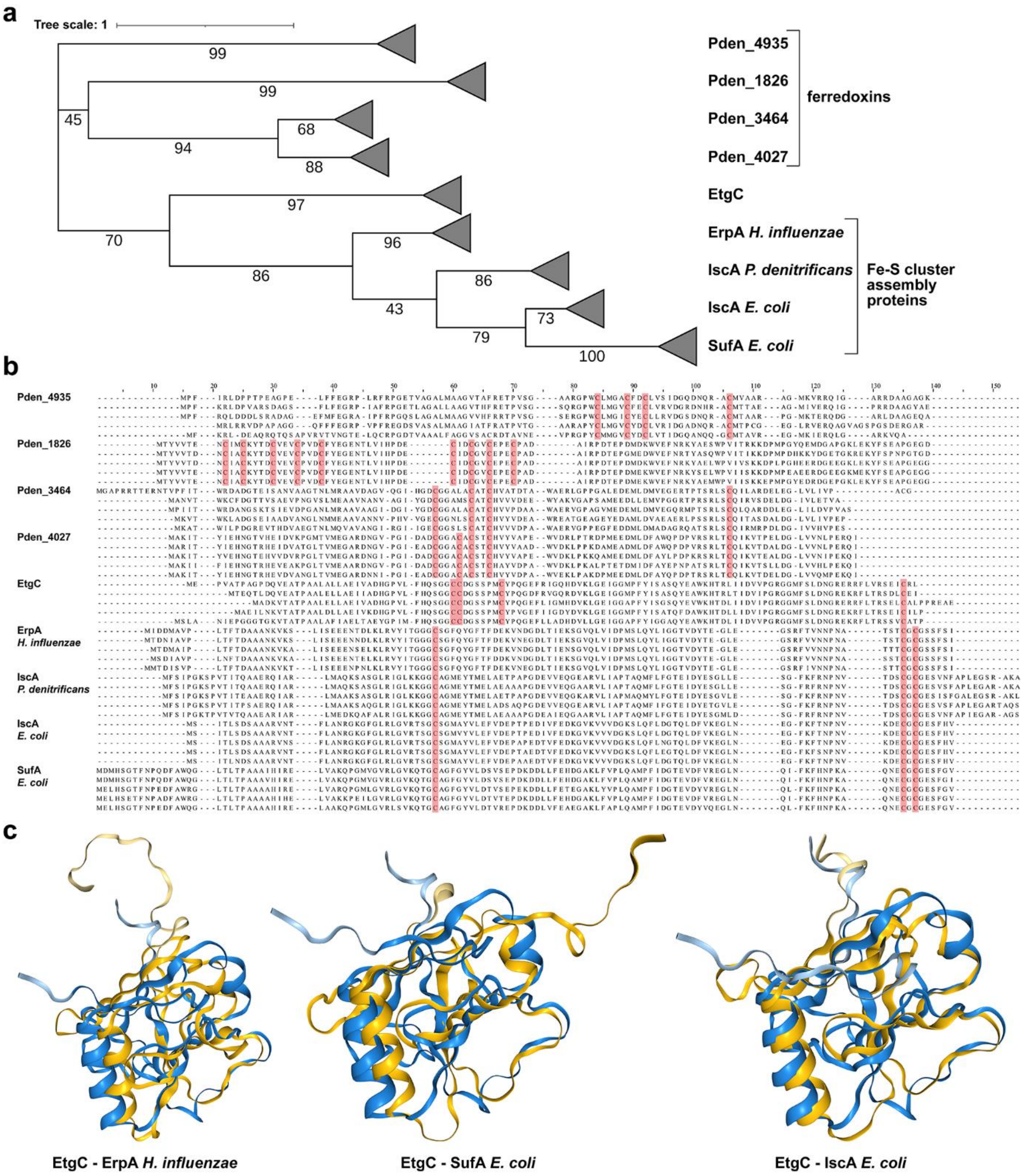

Supplementary Figure 10. Computational analysis of EtgC sequence and structure a, Maximum likelihood phylogenetic tree of EtgC homologs and related protein clades. Sequences of EtgC and its homologs constitute a distinct clade that forms a sister group to iron-sulfur (Fe-S) cluster assembly proteins. Bootstrap values are given on the respective nodes. **b**, Sequence alignment of EtgC homologs and related protein clades. Conserved cysteines are highlighted in red. **c**, Superimposed structures of EtgC (blue; predicted by AlphaFold (Jumper *et al.*[1]; https://alphafold.ebi.ac.uk/entry/A1B4L4) and three different Fe-S cluster assembly proteins (yellow; see Supplementary Table 3 for further information and PDB IDs).

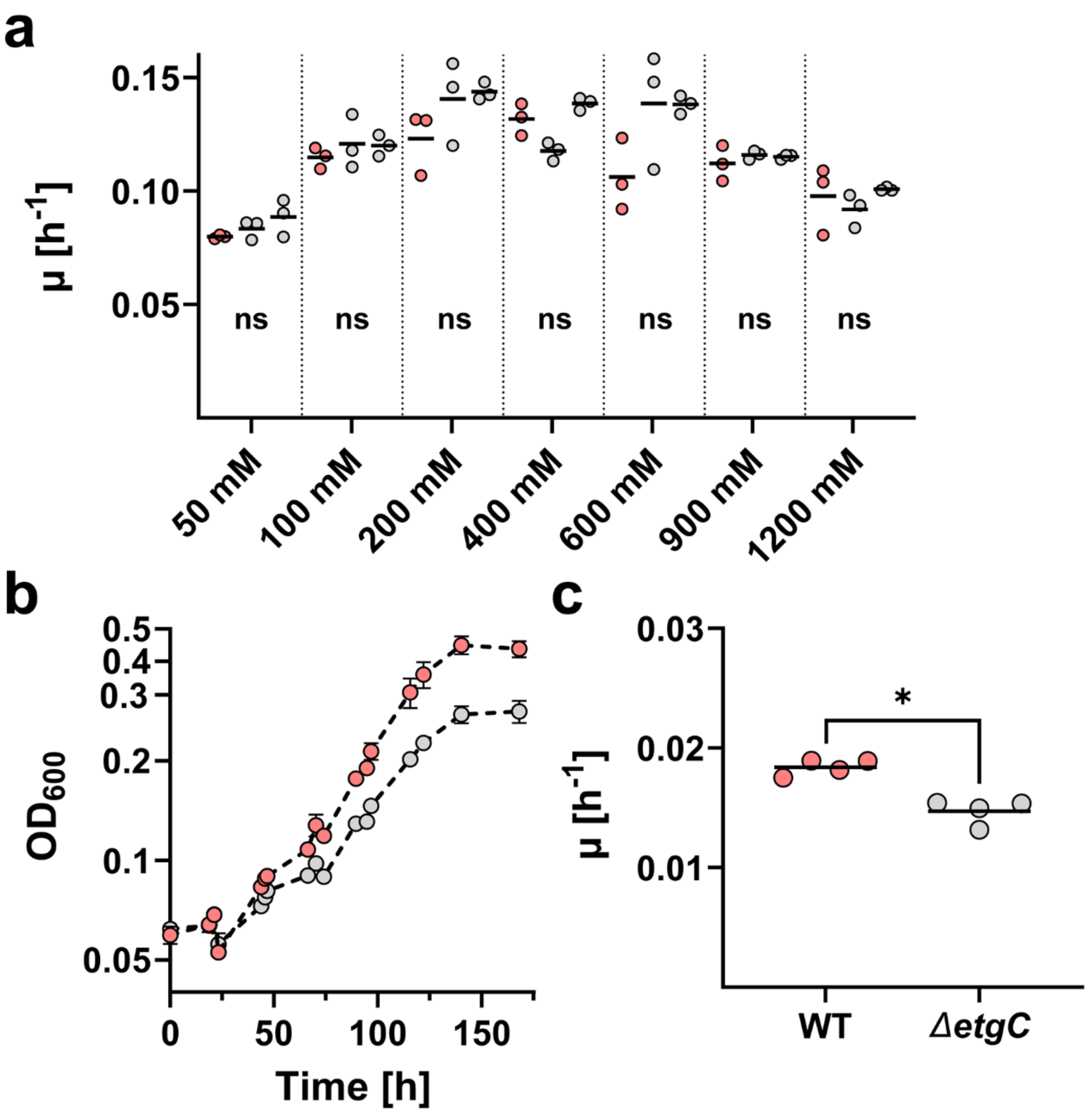

Supplementary Figure 11. Growth of *P. denitrificans* Δ*etgC* under aerobic and anaerobic conditions. **a**, Growth rates of *P. denitrificans* WT (red) and Δ*etgC* deletion strains (grey) grown aerobically in the presence of various concentrations of ethylene glycol. The results of $n = 3$ independently grown cultures are shown; black line represents the mean. ns: not significantly different from WT (*p* values for 50 mM: 0.2847, 0.2044; 100 mM: 0.4828, 0.2366; 200 mM: 0.2683, 0.1186; 400 mM: 0.0524, 0.2289; 600 mM: 0.1501, 0.0655; 900 mM: 0.4931, 0.5804; 1200 mM: 0.5861, 0.7650). **b**, Growth curves of the WT and Δ*etgC* strains in the presence of 30 mM ethylene glycol and 120 mM KNO_3 under anaerobic conditions. Data are the mean ± s.d. of $n = 4$ independently grown cultures. **c**, Growth rates calculated from this experiment. The results of $n = 4$ independently grown cultures are shown; black line represents the mean. *: significantly different from WT (*p* value: 0.0020). For **a** and **c**, results were compared using an unpaired *t*-test with Welch's correction in GraphPad Prism 8.1.1. Source data are provided as a Source Data file.

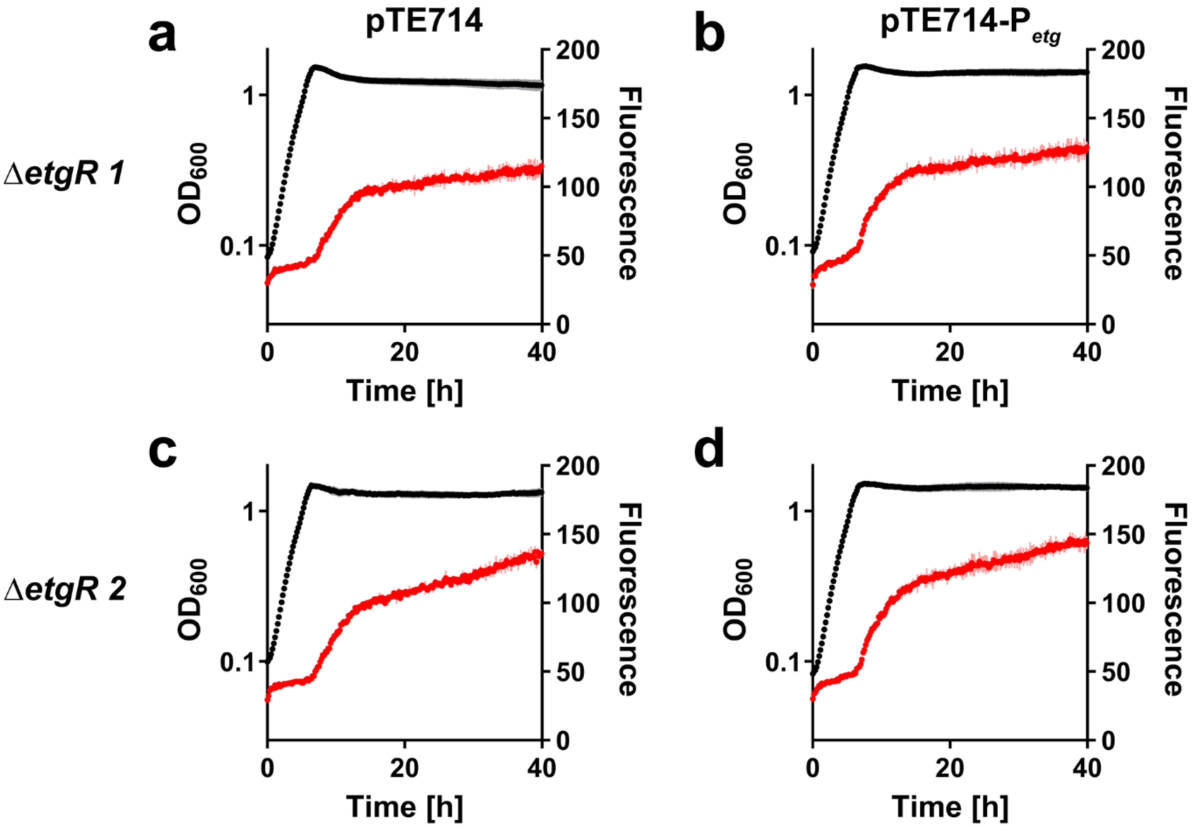

Supplementary Figure 12. Characterization of *P. denitrificans* Δ*etgR* promoter reporter strains grown on succinate and ethylene glycol. Growth (black) and fluorescence (red) of *P. denitrificans* Δ*etgR* 1 (**a, b**) and Δ*etgR* 2 (**c, d**) with pTE714 or pTE714-P_{etg} on 10 mM succinate and 60 mM ethylene glycol. Data are the mean ± s.d. of $n = 3$ independently grown cultures, with error bars shown in lighter colors. Source data are provided as a Source Data file.

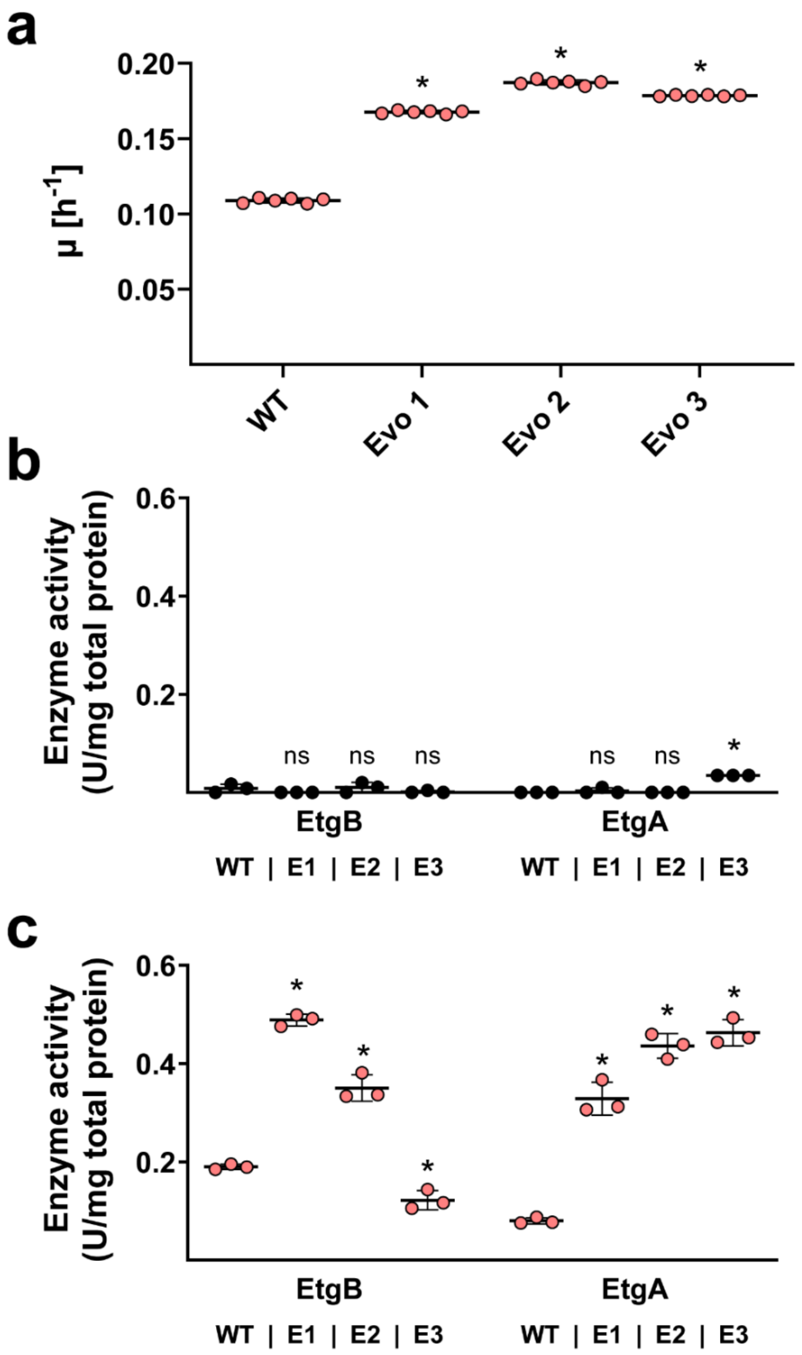

Supplementary Figure 13. Characterization of *P. denitrificans* strains evolved for improved growth on ethylene glycol. a, Growth rates of *P. denitrificans* WT and Evo1/2/3 on 200 mM ethylene glycol. The results of $n = 6$ independently grown cultures are shown. *: significantly different from WT (p values: 0.0001, 0.0001, 0.0001). **b** and **c,** Cell-free extract enzyme assays for EtgB and EtgA. Specific activities of EtgB (with 200 mM ethylene glycol) and EtgA (with 0.6 mM glycolaldehyde) in cell-free extracts of *P. denitrificans* WT and Evo1/2/3 grown on 30 mM succinate (**b**) or 60 mM ethylene glycol (**c**), as measured spectrophotometrically. Data are the mean ± s.d. of $n = 3$ independent experiments. ns: not significantly different from WT; *: significantly different from WT (p values for **b**: 0.2269, 0.8285, 0.2889, 0.4227, 0.9314, 0.0001; p values for **c**: 0.0001, 0.0073, 0.0204, 0.0047, 0.0009, 0.0009). For **a** to **c**, results were compared using an unpaired t-test with Welch's correction in GraphPad Prism 8.1.1. Source data are provided as a Source Data file.

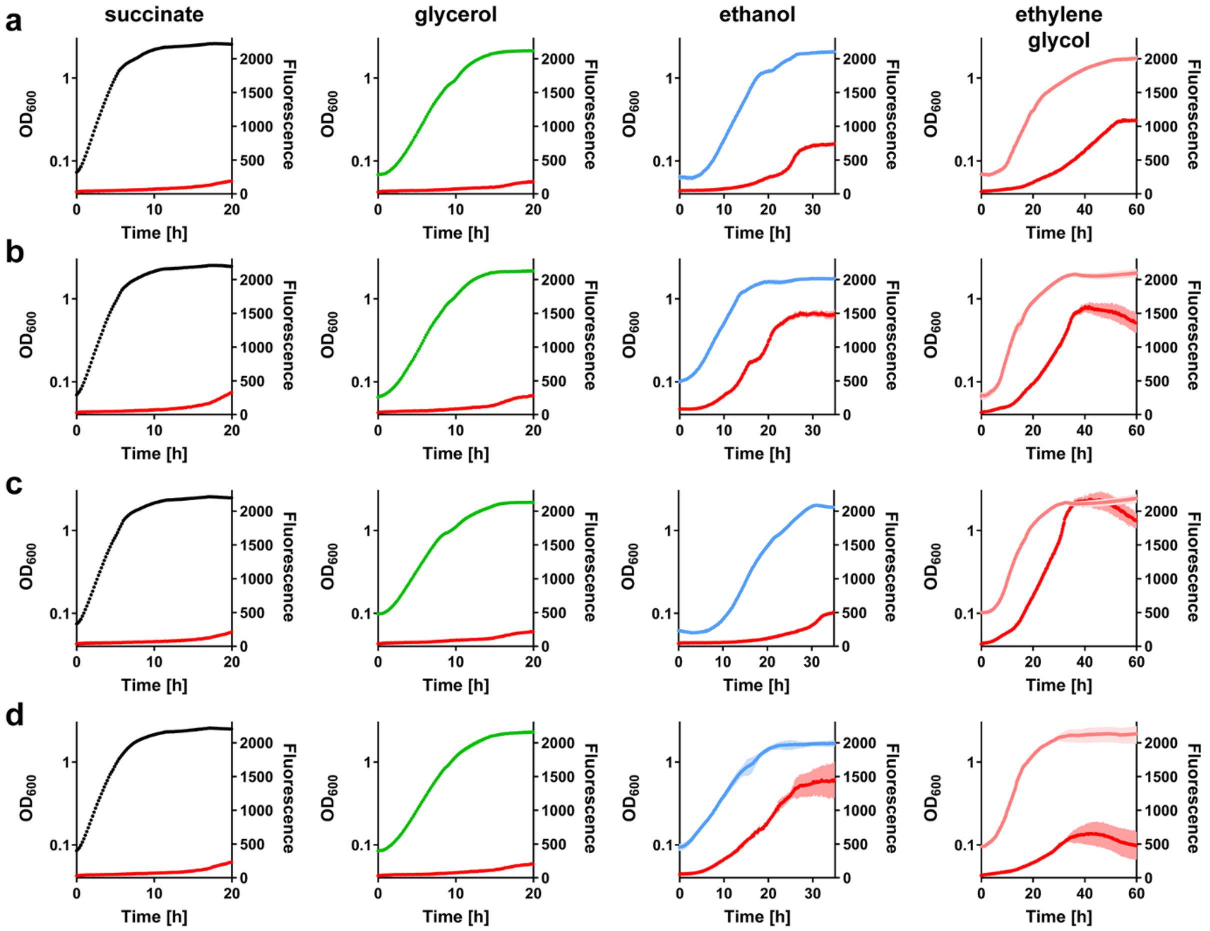

Supplementary Figure 14. Characterization of evolved *P. denitrificans* promoter reporter strains on different carbon sources. Growth and fluorescence (red) of *P. denitrificans* WT (**a**), Evo 1 (**b**), Evo 2 (**c**), and Evo 3 (**d**) with pTE714-P$_{etg}$ on different carbon sources. Data are the mean ± s.d. of n = 3 independently grown cultures, with error bars shown in lighter colors. Growth and fluorescence of negative control strains are shown in Supplementary Figure 15. Source data are provided as a Source Data file.

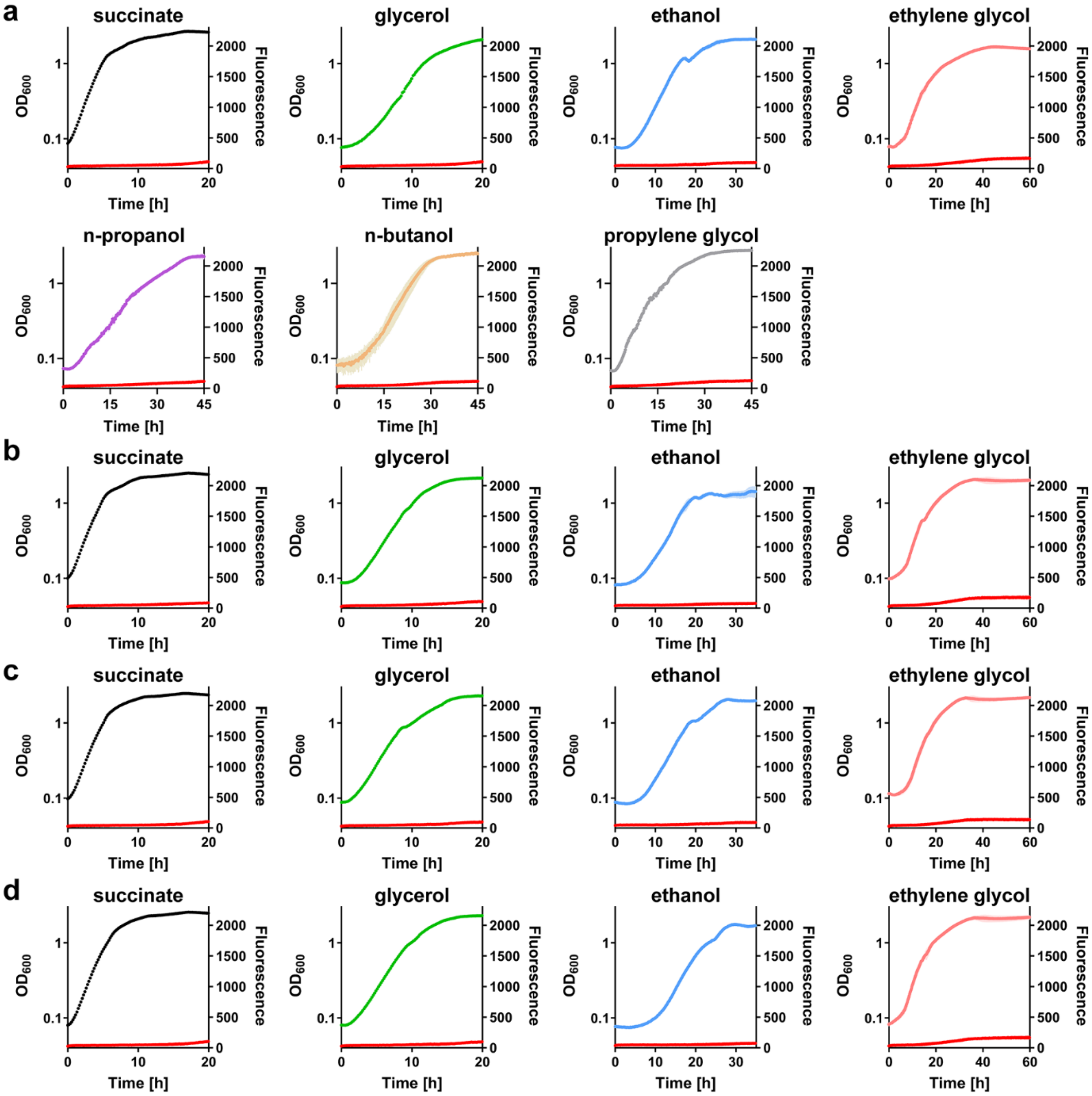

Supplementary Figure 15. Characterization of *P. denitrificans* promoter reporter strains on different carbon sources (negative controls). Growth and fluorescence (red) of *P. denitrificans* WT (**a**), Evo 1 (**b**), Evo 2 (**c**), and Evo 3 (**d**) with pTE714 (empty vector) on different carbon sources. Data are the mean ± s.d. of $n = 3$ independently grown cultures, with error bars shown in lighter colors. Source data are provided as a Source Data file.

Supplementary Table 1. Kinetic parameters of NAD-dependent alcohol dehydrogenases with ethylene glycol as substrate.

Enzyme	Organism	k_{cat} (s^{-1})	App. K_m (mM)	k_{cat}/K_m ($M^{-1}\,s^{-1}$)	Assay conditions	Reference
Alcohol dehydrogenase Gox0313	*Gluconobacter oxydans*	4.6 ± 0.2	964 ± 84	5	100 mM MOPS/KOH pH 7.8, 2 mM NAD^+, 37 °C	Scheffen *et al.*[2]
Alcohol dehydrogenase	*Pseudomonas aeruginosa*	4.5	> 200	< 23	100 mM Tris-HCl pH 8.8, 0.5 mM NAD^+, 40 °C	Levin *et al.*[3]
Alcohol dehydrogenase	*Homo sapiens*	0.8	290	3	100 mM glycine-NaOH pH 10, 2.4 mM NAD^+, 25 °C	Ditlow *et al.*[4]
Glycerol dehydrogenase	*Thermus thermophilus*	18.4	56	328	200 mM Tris-HCl pH 8.8, 8 mM NAD^+, 70 °C	Raghava and Gupta[5]
Glycerol dehydrogenase	*Klebsiella pneumoniae*	2.8 ± 0.2	93 ± 22	30	200 mM CHES pH 8.6, 10 mM NAD^+, 35 °C	Ko *et al.*[6]
Lactaldehyde reductase FucO	*Escherichia coli*	4.0 ± 0.1	51 ± 2	80	100 mM glycine-NaOH pH 10, 0.2 mM NAD^+, 30 °C	Blikstad and Widersten[7]
Alcohol dehydrogenase EtgB	*Paracoccus denitrificans*	7.2 ± 0.2	11 ± 1	651	100 mM glycine-NaOH pH 10, 2.4 mM NAD^+, 30 °C	This study
		5.5 ± 0.1	85 ± 6	65	100 mM potassium phosphate pH 7.5, 2.4 mM NAD^+, 30 °C	

Supplementary Table 2. Cryo-EM data collection, refinement and validation statistics.

	EtgA	EtgB
**Data collection**		
Microscope	Titan Krios	Titan Krios
Voltage (kV)	300	300
Magnification	105,000x	105,000x
Detector - GIF	Gatan K3 - Bioquantum	Gatan K3 - Bioquantum
Data collection software	EPU	EPU
Electron exposure ($e^-/Å^2$)	50	50
Defocus range (μm)	0.8 - 2.2	0.8 - 2.2
Pixel size (Å)	0.836	0.836
**Data processing**		
Software	Relion 5	Relion 5
Number of micrographs	6697	5412
Final number of particles	185597	206685
Symmetry imposed	C1	C1
Map resolution (Å)	3.1	3.0
FSC threshold	0.143	0.143
**Model refinement**		
Software	Phenix 1.18.2	Phenix 1.18.2
Map correlation coefficient	0.78	0.79
Model composition		
Number of chains	4	4
Non-hydrogen atoms	12756	9910
Protein residues	1654	1346
Ligands	2	8
R.M.S. deviations		
Bond lengths (Å)	0.006	0.008
Bond angles (°)	0.720	0.835
**Validation**		
MolProbity score	2.12	2.07
Clashscore	14.5	15.06
Rotamer outliers (%)	0	0
Ramachandran plot		
Favored (%)	92.98	94.29
Allowed (%)	7.02	5.71
Disallowed (%)	0	0
**Data availability**		
EMDB entry	EMD-50550	EMD-50545
PDB entry	9FM9	9FLZ

Supplementary Table 3. Structural similarity of EtgA, EtgB, and EtgC to other proteins. The analysis was performed using Foldseek (van Kempen *et al.*[8]; search.foldseek.com). For EtgA and EtgB, the structures determined in this study were used as query; for EtgC, the structure predicted by AlphaFold (Jumper *et al.*[1]; https://alphafold.ebi.ac.uk/entry/A1B4L4) was used as query.

	**Enzyme**	**Organism**	**PDB ID**	**Sequence identity (%)**	**RMSD (Å)**	**Reference**
**EtgA**	Mitochondrial aldehyde dehydrogenase 2 (ALDH2)	*Homo sapiens*	4FR8	42.7	1.67	Lang *et al.*[9]
	Aldehyde dehydrogenase	*Burkholderia cenocepacia*	4O6R	40.7	1.35	Unpublished
	Aldehyde dehydrogenase	*Bacillus cereus*	4QF6	40.7	1.27	Unpublished
	Aldehyde dehydrogenase PnpE	*Pseudomonas sp. WBC-3*	4GO3	39.4	1.34	Unpublished
	Indole-3-acetaldehyde dehydrogenase	*Pseudomonas syringae*	5IUV	39.3	1.63	McClerklin *et al.*[10]
**EtgB**	Alcohol dehydrogenase	*Brucella suis*	3MEQ	87.2	0.59	Unpublished
	Alcohol dehydrogenase	*Halomonas elongata*	6Z42	75.8	0.99	Contente *et al.*[11]
	Alcohol dehydrogenase FurX	*Cupriavidus pinatubonensis*	3S2I	74.1	1.13	Unpublished
	Alcohol dehydrogenase	*Moraxella sp. TAE123*	4Z6K	72.1	0.92	Petratos *et al.*[12]
	Alcohol dehydrogenase	*Pseudomonas aeruginosa*	1LLU	71.9	1.21	Levin *et al.*[3]
**EtgC**	Iron-sulfur cluster insertion protein ErpA	*Haemophilus influenzae*	2APN	16.2	5.48	Unpublished
	Iron-sulfur cluster assembly protein SufA	*Escherichia coli*	2D2A	14.2	7.62	Wada *et al.*[13]
	Iron-sulfur cluster assembly protein IscA	*Escherichia coli*	1S98	14	5.77	Cupp-Vickery *et al.*[14]

Supplementary Table 4. Metal content of EtgC as determined via ICP-OES. Data are mean ± s.d. from *n* = 3 independent experiments. Limit of detection (LoD): 2 * 10^{-3} metal atoms per EtgC monomer.

	**metal atoms per EtgC monomer**
Cobalt	below LoD
Copper	(2.84 ± 0.04) * 10^{-3}
Iron	(5.90 ± 0.60) * 10^{-3}
Manganese	(1.36 ± 0.02) * 10^{-3}
Zinc	(20.90 ± 2.75) * 10^{-3}

Supplementary Table 5. Strains used in this study.

**Strain**	**Genotype or relevant features**[a]	**Source or reference**
E. coli DH5□	*supE44*, *ΔlacU169* (*Φ80lacZDM15*), *hsdR17*, *recA1*, *endA1*, *gyrA96*, *thi*-1, *relA1*	Thermo Fisher Scientific, Waltham, USA
E. coli ST18	*pro*, *thi*, *hsdR1*,Tp^R, Sm^R; chromosome::RP4-2, Tc::Mu-Kan::Tn7/λpir, λpir, Δ*hemA*	Thoma and Schobert[15]
E. coli BL21 AI	*ompT*, *gal*, *dcm*, *lon*, *hsdS*$_B$($r_B^- m_B^-$), [*malB*$^+$]$_{K-12}$(λ^S), *araB*::*T7RNAP-tetA*	Thermo Fisher Scientific, Waltham, USA
E. coli K-12 W3110	F$^-$, λ$^-$, IN(*rrnD-rrnE*)1	Bachmann[16]
P. denitrificans DSM 413	WT strain	Beijerinck and Minkman[17]
P. denitrificans DSM 413 Δ*etgR* 1	Δ*etgR*; Km^R (orientation 1)	This work
P. denitrificans DSM 413 Δ*etgR* 2	Δ*etgR*; Km^R (orientation 2)	This work
P. denitrificans DSM 413 Δ*etgA* 1	Δ*etgA*; Km^R (orientation 1)	This work
P. denitrificans DSM 413 Δ*etgA 2*	Δ*etgA*; Km^R (orientation 2)	This work
P. denitrificans DSM 413 Δ*etgB* 1	Δ*etgB*; Km^R (orientation 1)	This work
P. denitrificans DSM 413 Δ*etgB 2*	Δ*etgB*; Km^R (orientation 2)	This work
P. denitrificans DSM 413 Δ*etgC* 1	Δ*etgC*; Km^R (orientation 1)	This work
P. denitrificans DSM 413 Δ*etgC* 2	Δ*etgC*; Km^R (orientation 2)	This work
P. denitrificans DSM 413 Δ*mxaF 1*	Δ*mxaF*; Km^R (orientation 1)	This work
P. denitrificans DSM 413 Δ*mxaF 2*	Δ*mxaF*; Km^R (orientation 2)	This work
P. denitrificans DSM 413 Evo 1	evolved on ethylene glycol; see Table 2	This work
P. denitrificans DSM 413 Evo 2	evolved on ethylene glycol; see Table 2	This work
P. denitrificans DSM 413 Evo 3	evolved on ethylene glycol; see Table 2	This work

[a] Km^R, kanamycin resistance; Tp^R, trimethoprim resistance; Sm^R, streptomycin resistance

Supplementary Table 6. Plasmids used in this study.

Plasmid	Relevant features[a]	Source or reference
pET16b	*E. coli* expression vector, T7 promoter, Amp^R	Merck Chemicals GmbH, Darmstadt, Germany
pET16b-EtgA	expression vector for N-terminally His-tagged EtgA, Amp^R	This work
pET16b-EtgA Y464G	expression vector for N-terminally His-tagged EtgA Y464G, Amp^R	This work
pET16b-EtgB	expression vector for N-terminally His-tagged EtgB, Amp^R	This work
pET16b-EtgB T44S	expression vector for N-terminally His-tagged EtgB T44S, Amp^R	This work
pET16b-EtgB H47N	expression vector for N-terminally His-tagged EtgB H47N, Amp^R	This work
pET16b-EtgB T44S H47N	expression vector for N-terminally His-tagged EtgB T44S H47N, Amp^R	This work
pET16b-EtgC	expression vector for N-terminally His-tagged EtgC, Amp^R	This work
pZ-ASS-mCherry	*E. coli* expression vector with p15A origin and constitutive strong promoter, $Strep^R$	Wenk *et al.*[18]
pZ-ASS-EtgB	expression vector for EtgB, $Strep^R$	This work
pREDSIX	mobilizable, high-copy-number cloning and mutagenesis vector; Amp^R	Ledermann *et al.*[19]
pRGD-KmR	donor vector for resistance gene, *aphII* in polylinker; Amp^R, Km^R	Ledermann *et al.*[19]
pREDSIX-*etgR-1*	knockout vector for the *etgR* gene in *P. denitrificans* DSM 413; Km^R (orientation 1)	This work
pREDSIX-*etgR-2*	knockout vector for the *etgR* gene in *P. denitrificans* DSM 413; Km^R (orientation 2)	This work
pREDSIX-*etgA-1*	knockout vector for the *etgA* gene in *P. denitrificans* DSM 413; Km^R (orientation 1)	This work
pREDSIX-*etgA-2*	knockout vector for the *etgA* gene in *P. denitrificans* DSM 413; Km^R (orientation 2)	This work
pREDSIX-*etgB-1*	knockout vector for the *etgB* gene in *P. denitrificans* DSM 413; Km^R (orientation 1)	This work
pREDSIX-*etgB-2*	knockout vector for the *etgB* gene in *P. denitrificans* DSM 413; Km^R (orientation 2)	This work
pREDSIX-*etgC-1*	knockout vector for the *etgC* gene in *P. denitrificans* DSM 413; Km^R (orientation 1)	This work
pREDSIX-*etgC-2*	knockout vector for the *etgC* gene in *P. denitrificans* DSM 413; Km^R (orientation 2)	This work
pREDSIX-*mxaF-1*	knockout vector for the *mxaF* gene in *P. denitrificans* DSM 413; Km^R (orientation 1)	This work
pREDSIX-*mxaF-2*	knockout vector for the *mxaF* gene in *P. denitrificans* DSM 413; Km^R (orientation 2)	This work
pTE714	promoter probe vector for Alphaproteobacteria containing a RBS and mCherry; Tc^R	Schada von Borzyskowski *et al.*[20]
pTE714_2365/2366_ig	promoter probe vector containing mCherry under control of the promoter located in the intergenic region between Pden_2365 and Pden_2366; Tc^R	This work

[a] Km^R, kanamycin resistance; Amp^R, ampicillin resistance; Tc^R, tetracycline resistance; $Strep^R$, streptomycin resistance

Supplementary Table 7. Primers used in this study.

Target	Name	Sequence[a]	Cut site
Pden_*etgA*	etgA_16b_fw	5’-CTAGAGT**CATATG**CCGAACGACCAGACG-3’	*Nde*I
Pden_*etgA*	etgA_16b_rv	5’-GACTACT**GGATCC**TCAGAAGAAGCCCAGCTTC-3’	*Bam*HI
Pden_*etgB*	etgB_16b_fw	5’-CTCGTAC**ATTAAT**ATGGCCAAAACCATGAAAGCCG-3’	*Ase*I
Pden_*etgB*	etgB_16b_rv	5’-CATTACT**CTCGAG**TCAGCCCGCCATGTCCAGCAC-3’	*Xho*I
Pden_*etgC*	etgC_16b_fw	5’-GACTAG**CATATG**GAACCCGTCGCCACCC-3’	*Nde*I
Pden_*etgC*	etgC_16b_rv	5’-GTTTAC**GGATCC**TCAGAGCCGGCAGATCTCGGAC-3’	*Bam*HI
Pden_*etgA*	Y464G_fw	5’-CCACGCCGGCCCGGCCCATGCG-3’	---
Pden_*etgA*	Y464G_rv	5’-CCGGGCCGGCGTGGTAGCAGTTGG-3’	---
Pden_*etgB*	T44S_fw	5’-TGCCATAGCGACCTGCACGCGGCCGAGGGC-3’	---
Pden_*etgB*	T44S_rv	5’-GGTCGCTATGGCAGACGCCCGAGGCCTG-3’	---
Pden_*etgB*	H47N_fw	5’-ACCTGAACGCGGCCGAGGGCGACTG-3’	---
Pden_*etgB*	H47N_rv	5’-CGGCCGCGTTCAGGTCGGTATGGCAGA-3’	---
Pden_*etgB*	T44S_H47N_fw	5’-GCCATTCGGACCTGAACGCGGCC-3’	---
Pden_*etgB*	T44S_H47N_rv	5’-TCAGGTCCGAATGGCAGACGCCCG-3’	---
Pden_*etgB*	etgB_ZA_fw	5’-AGAGGCAAGAATGGCCAAAACCATGAAAGCC-3’	---
Pden_*etgB*	etgB_ZA_rv	5’-CCGCGCTAGCTCAGCCCGCCATGTCCAG-3’	---
pZ-ASS	pZ-ASS_fw	5’-GGCGGGCTGAGCTAGCGCGGCCGCTGCA-3’	---
pZ-ASS	pZ-ASS_rv	5’-TTTTGGCCATTCTTGCCTCTTAACTTTAAAGTTAAACAAAATTAT-3’	---
pZ-ASS-EtgB	etgB_ZAseq_fw	5’-CGCGTTGTTTCATCAAGCCT-3’	---
pZ-ASS-EtgB	etgB_ZAseq_rv	5’-GGGTTCGTGCATACAGTCCA-3’	---
Pden_*etgR*_up	etgR_up_fw	5’-GGTCTGACAGGTTTAAACTCTAGACGGATCGCCGAGCGCATCGCGC-3’	---
Pden_*etgR*_up	etgR_up_rv	5’-GCCCCGATGTGTT**CATATG**CGAAGCACCCCGGCCTTTC-3’	*Nde*I
Pden_*etgR*_down	etgR_down_fw	5’-GCTTCG**CATATG**AACACATCGGGGCAGCGGCGCG-3’	*Nde*I
Pden_*etgR*_down	etgR_down_rv	5’-CTTAAGGCTAGCATGCATCCTAGGCAGCACGCCCGGCGGCAGCAGG-3’	---
Pden_Δ*etgR*	etgR_seq_fw	5’-GCCATCGACAACCTCAGGAA-3’	---
Pden_Δ*etgR*	etgR_seq_rv	5’-GATCATGGTGTCGCTTTCGC-3’	---
Pden_*etgA*_up	etgA_up_fw	5’-GACAGGTTTAAACTCTAGACTGTGGCGCGGCATGGACT-3’	---
Pden_*etgA*_up	etgA_up_rv	5’-GGCCATTCCA**GGTACC**GATGTTCCTCCTGTCATTC-3’	*Kpn*I
Pden_*etgA*_down	etgA_down_fw	5’-CATC**GGTACC**TGGAATGGCCGGGCGCAT-3’	*Kpn*I
Pden_*etgA*_down	etgA_down_rv	5’-GGCTAGCATGCATCCTAGGCGTTCGGTCGCGGCGTTCAC-3’	---
Pden_Δ*etgA*	etgA_seq_fw	5’-TGCTTCGTTGCGGGCGCTTCAC-3’	---
Pden_Δ*etgA*	etgA_seq_rv	5’-ATTCCTGCAGGTCCAGCCGCGTG-3’	---
Pden_*etgB*_up	etgB_up_fw	5’-GACAGGTTTAAACTCTAGACGGCGCTGGAAGGCTTTAC-3’	---
Pden_*etgB*_up	etgB_up_rv	5’-GGTTCCATGC**CATATG**GAGAGGGCACTCC-3’	*Nde*I
Pden_*etgB*_down	etgB_down_fw	5’-TCTC**CATATG**GCATGGAACCCGTCGCCA-3’	*Nde*I
Pden_*etgB*_down	etgB_down_rv	5’-GGCTAGCATGCATCCTAGGCGTCAATTCGCTGTCCATGCTGC-3’	---
Pden_Δ*etgB*	etgB_seq_fw	5’-ACTGCGTGGTGCTGAAACCGGC-3’	---
Pden_Δ*etgB*	etgB_seq_rv	5’-CGCGTTCGCGTTTCCGGCTGAAC-3’	---
Pden_*etgC*_up	etgC_up_fw	5’-GGTCTGACAGGTTTAAACTCTAGACCGGCCATTGCCGGCATTGCCT-3’	---
Pden_*etgC*_up	etgC_up_rv	5’-CCCGTGCCGCGG**GGTACC**GCTCAGCCCGCCATG-3’	*Kpn*I
Pden_*etgC*_down	etgC_down_fw	5’-GCTGAGC**GGTACC**CCGCGGCACGGGCGCACGGCC-3’	*Kpn*I
Pden_*etgC*_down	etgC_down_rv	5’CTTAAGGCTAGCATGCATCCTAGGCTGAAGACCCCCTCGCGGCTGATCGGCATGATCCAG-3’	---
Pden_Δ*etgC*	etgC_seq_fw	5’-GGACCTGCAGGAATCGCTG-3’	---
Pden_Δ*etgC*	etgC_seq_rv	5’-CTGGTCAATTCGCTGTCCATG-3’	---
Pden_*mxaF*_up	mxaF_up_fw	5’-GACAGGTTTAAACTCTAGACGACGCGATATTGCTGGGC-3’	---
Pden_*mxaF* _up	mxaF_up_rv	5’-GCCCGCCGCG**CATATG**AGCGATCTCGCTC-3’	*Nde*I
Pden_*mxaF* _down	mxaF_down_fw	5’-CGCT**CATATG**GGCCCGCCCTTCCTTC-3’	*Nde*I
Pden_*mxaF* _down	mxaF_down_rv	5’-CTGGTCAATTCGCTGTCCATG-3’	---
Pden_Δ*mxaF*	mxaF_seq_fw	5’-CAGGCACAGCGACAGGCTGAC-3’	---
Pden_Δ*mxaF*	mxaF_seq_rv	5’-GGCTAGCATGCATCCTAGGCCCGTCCGAGCGTTCGATTTC-3’	---
Pden_2365/2366_ig	2365/2366_ig_fw	5’-TTGACA**GAATTC**CGAGGGCAATGTCTCGGC-3’	*Eco*RI
Pden_2365/2366_ig	2365/2366_ig_rv	5’-ACTCAA**TCTAGA**TCAGATGTTCCTCCTGTCATTCCGGCT-3’	*Xba*I

[a] Nucleotides in bold and underlined are recognition sites for endonuclease restriction enzymes.

Supplementary references

1. Jumper J. et al. Highly accurate protein structure prediction with AlphaFold. *Nature* **596**, 583-589 (2021).
2. Scheffen M. et al. A new-to-nature carboxylation module to improve natural and synthetic CO2 fixation. *Nature Catalysis* **4**, 105-115 (2021).
3. Levin I., Meiri G., Peretz M., Burstein Y., Frolow F. The ternary complex of *Pseudomonas aeruginosa* alcohol dehydrogenase with NADH and ethylene glycol. *Protein Science* **13**, 1547-1556 (2004).
4. Ditlow C. C., Holmquist B., Morelock M. M., Vallee B. L. Physical and enzymic properties of a class II alcohol dehydrogenase isozyme of human liver: pi-ADH. *Biochemistry* **23**, 6363-6368 (1984).
5. Raghava S., Gupta M. N. Purification and characterization of an alcohol dehydrogenase with an unusual specificity towards glycerol from *Thermus thermophilus*. *Bioresource Technology* **101**, 2554-2557 (2010).
6. Ko G. S., Nguyen Q. T., Kim D. H., Yang J. K. Biochemical and molecular characterization of glycerol dehydrogenase from *Klebsiella pneumoniae*. *J Microbiol Biotechnol* **30**, 271-278 (2020).
7. Blikstad C., Widersten M. Functional characterization of a stereospecific diol dehydrogenase, FucO, from *Escherichia coli*: Substrate specificity, pH dependence, kinetic isotope effects and influence of solvent viscosity. *Journal of Molecular Catalysis B: Enzymatic* **66**, 148-155 (2010).
8. van Kempen M. et al. Fast and accurate protein structure search with Foldseek. *Nat Biotechnol* **42**, 243-246 (2024).
9. Lang B. S. et al. Vascular bioactivation of nitroglycerin by aldehyde dehydrogenase-2. *Journal of Biological Chemistry* **287**, 38124-38134 (2012).
10. McClerklin S. A., Lee S. G., Harper C. P., Nwumeh R., Jez J. M., Kunkel B. N. Indole-3-acetaldehyde dehydrogenase-dependent auxin synthesis contributes to virulence of *Pseudomonas syringae* strain DC3000. *PLOS Pathogens* **14**, e1006811 (2018).
11. Contente M. L. et al. Uncommon overoxidative catalytic activity in a new halo-tolerant alcohol dehydrogenase. *ChemCatChem* **12**, 5679-5685 (2020).
12. Petratos K. et al. Structure and Dynamics of a Thermostable alcohol dehydrogenase from the antarctic psychrophile *Moraxella* sp. TAE123. *ACS Omega* **5**, 14523-14534 (2020).
13. Wada K., Hasegawa Y., Gong Z., Minami Y., Fukuyama K., Takahashi Y. Crystal structure of *Escherichia coli* SufA involved in biosynthesis of iron–sulfur clusters: Implications for a functional dimer. *FEBS Letters* **579**, 6543-6548 (2005).
14. Cupp-Vickery J. R., Silberg J. J., Ta D. T., Vickery L. E. Crystal structure of IscA, an iron-sulfur cluster assembly protein from *Escherichia coli*. *Journal of Molecular Biology* **338**, 127-137 (2004).
15. Thoma S., Schobert M. An improved *Escherichia coli* donor strain for diparental mating. *FEMS Microbiology Letters* **294**, 127-132 (2009).
16. Bachmann B. J. Pedigrees of some mutant strains of *Escherichia coli* K-12. *Bacteriological Reviews* **36**, 525-557 (1972).
17. Beijerinck M. W., Minkman D. C. J. Bildung und verbrauch von stickoxydul durch bakterien. *Zentralbl Bakteriol Naturwiss* **25**, 30–63 (1910).

18. Wenk S., Yishai O., Lindner S. N., Bar-Even A. Chapter Twelve - An Engineering Approach for Rewiring Microbial Metabolism. In: *Methods in Enzymology* (ed Scrutton N). Academic Press (2018).

19. Ledermann R., Strebel S., Kampik C., Fischer H. M. Versatile vectors for efficient mutagenesis of *Bradyrhizobium diazoefficiens* and other Alphaproteobacteria. *Appl Environ Microbiol* **82**, 2791-2799 (2016).

20. Schada von Borzyskowski L. et al. Multiple levels of transcriptional regulation control glycolate metabolism in *Paracoccus denitrificans*. *Mbio* **15**, e01524-01524 (2024).
