# Supplementary material for: NAD-dependent dehydrogenases enable efficient growth of Paracoccus denitrificans on the PET monomer ethylene glycol
Source: Nat Commun. 2025 Jul 1;16:5845. doi: 10.1038/s41467-025-61056-x (PMC12214560; doi:10.1038/s41467-025-61056-x)
Supplement: Supplementary file 3 — Description of Additional Supplementary Files [file 41467_2025_61056_MOESM3_ESM.pdf]

Description of Supplementary Data Files

File Name: Supplementary Data File 1

Description: Table of bacterial genomes that contain homologs of EtgR/A/B/C

File Name: Supplementary Data File 2

Description: List of sequences of homologs of EtgR/A/B/C and associated metadata

File Name: Supplementary Data File 3

Description: Zip file containing all Genbank files that were used to generate Figure 7c (EtgRABC)

File Name: Supplementary Data File 4

Description: Zip file containing all Genbank files that were used to generate Figure 7c (EtgRAC)

File Name: Supplementary Data File 5

Description: List of sequences that were used to generate Supplementary Figure 10a

File Name: Supplementary Data File 6

Description: List of sequences that were used to generate Supplementary Figure 10b
