# Supplementary material for: NAD-dependent dehydrogenases enable efficient growth of Paracoccus denitrificans on the PET monomer ethylene glycol
Source: Nat Commun. 2025 Jul 1;16:5845. doi: 10.1038/s41467-025-61056-x (PMC12214560; doi:10.1038/s41467-025-61056-x)
Supplement: Supplementary file 10 — Reporting Summary [file 41467_2025_61056_MOESM10_ESM.pdf]

nature portfolio

Corresponding author(s): Lennart Schada von Borzyskowski

Last updated by author(s): Jun 5, 2025

Reporting Summary

Nature Portfolio wishes to improve the reproducibility of the work that we publish. This form provides structure for consistency and transparency in reporting. For further information on Nature Portfolio policies, see our Editorial Policies and the Editorial Policy Checklist.

Statistics

For all statistical analyses, confirm that the following items are present in the figure legend, table legend, main text, or Methods section.

n/a	Confirmed	
☐	☒	The exact sample size (*n*) for each experimental group/condition, given as a discrete number and unit of measurement
☐	☒	A statement on whether measurements were taken from distinct samples or whether the same sample was measured repeatedly
☐	☒	The statistical test(s) used AND whether they are one- or two-sided<br>*Only common tests should be described solely by name; describe more complex techniques in the Methods section.*
☒	☐	A description of all covariates tested
☒	☐	A description of any assumptions or corrections, such as tests of normality and adjustment for multiple comparisons
☐	☒	A full description of the statistical parameters including central tendency (e.g. means) or other basic estimates (e.g. regression coefficient) AND variation (e.g. standard deviation) or associated estimates of uncertainty (e.g. confidence intervals)
☐	☒	For null hypothesis testing, the test statistic (e.g. *F*, *t*, *r*) with confidence intervals, effect sizes, degrees of freedom and *P* value noted<br>*Give P values as exact values whenever suitable.*
☒	☐	For Bayesian analysis, information on the choice of priors and Markov chain Monte Carlo settings
☒	☐	For hierarchical and complex designs, identification of the appropriate level for tests and full reporting of outcomes
☒	☐	Estimates of effect sizes (e.g. Cohen's *d*, Pearson's *r*), indicating how they were calculated

Our web collection on statistics for biologists contains articles on many of the points above.

Software and code

Policy information about availability of computer code

Data collection	Data collection and analysis was performed using publicly available software as detailed in citations included in the manuscript and SI. Cary WinUV 5.0.0.999; GraphPad Prism 8.1.1; Tecan iControl; Excel; RELION-5.0; MotionCor2; CTFFIND-4; Topaz; AlphaFold 2; Coot; Phenix; ChimeraX; PyMOL; Progenesis QI 2.0; MASCOT 2.5; SafeQuant 2.2.2; blastp; CAGECAT/clinker; Foldseek
Data analysis	Data collection and analysis was performed using publicly available software as detailed in citations included in the manuscript and SI. Cary WinUV 5.0.0.999; GraphPad Prism 8.1.1; Tecan iControl; Excel; RELION-5.0; MotionCor2; CTFFIND-4; Topaz; AlphaFold 2; Coot; Phenix; ChimeraX; PyMOL; Progenesis QI 2.0; MASCOT 2.5; SafeQuant 2.2.2; blastp; CAGECAT/clinker; Foldseek

For manuscripts utilizing custom algorithms or software that are central to the research but not yet described in published literature, software must be made available to editors and reviewers. We strongly encourage code deposition in a community repository (e.g. GitHub). See the Nature Portfolio guidelines for submitting code & software for further information.

Data

Policy information about availability of data

All manuscripts must include a data availability statement. This statement should provide the following information, where applicable:

- Accession codes, unique identifiers, or web links for publicly available datasets
- A description of any restrictions on data availability
- For clinical datasets or third party data, please ensure that the statement adheres to our policy

Genome sequencing data of evolved P. denitrificans isolates are available in the NCBI SRA database with the identifier PRJNA1126100 (https://www.ncbi.nlm.nih.gov/sra/?term=PRJNA1126100). Mass spectrometry proteomics data are available via ProteomeXchange with the identifier PXD060720 (http://proteomecentral.proteomexchange.org/cgi/GetDataset?ID=PXD060720). Cryo-EM maps for EtgA and EtgB are deposited in the EMDB under codes EMD-50550 (https://www.ebi.ac.uk/pdbe/entry/emdb/EMD-50550) and EMD-50545 (https://www.ebi.ac.uk/pdbe/entry/emdb/EMD-50545). Atomic coordinates of EtgA and EtgB are deposited in the PDB under codes 9FM9 (http://doi.org/10.2210/pdb9FM9/pdb) and 9FLZ (http://doi.org/10.2210/pdb9FLZ/pdb). All other relevant data are available in this article and its Supplementary Information files. Source data for Figures 2, 3, 6 and Supplementary Information Figures 1, 2, 3, 4, 5, 6, 9, 10, 11, 12, 13 are provided with this paper.

Research involving human participants, their data, or biological material

Policy information about studies with human participants or human data. See also policy information about sex, gender (identity/presentation), and sexual orientation and race, ethnicity and racism.

Reporting on sex and gender	No research involving human participants, their data, or biological material is reported in this manuscript.
Reporting on race, ethnicity, or other socially relevant groupings	No research involving human participants, their data, or biological material is reported in this manuscript.
Population characteristics	No research involving human participants, their data, or biological material is reported in this manuscript.
Recruitment	No research involving human participants, their data, or biological material is reported in this manuscript.
Ethics oversight	No research involving human participants, their data, or biological material is reported in this manuscript.

Note that full information on the approval of the study protocol must also be provided in the manuscript.

Field-specific reporting

Please select the one below that is the best fit for your research. If you are not sure, read the appropriate sections before making your selection.

☒ Life sciences ☐ Behavioural & social sciences ☐ Ecological, evolutionary & environmental sciences

For a reference copy of the document with all sections, see nature.com/documents/nr-reporting-summary-flat.pdf

Life sciences study design

All studies must disclose on these points even when the disclosure is negative.

Sample size	No sample size calculation was performed. The common rationale was to have a sample size that allows us to calculate a standard deviation. All Michaelis-Menten plots for the kinetic characterization of enzymes include at least 18 data points (three independent experiments at 6 or more different substrate concentrations). Controls verifying same levels of specific activities between different enzyme preparations were performed routinely.<br>Three independent experiments (= assays in independent cuvettes) were conducted for the determination of enzyme activities in vitro, both with purified enzymes and in P. denitrificans cell extracts.<br>Three biological replicates (= independent cultures) of P. denitrificans were used to generate cell-free extracts for determination of enzyme activities.<br>Three to six biological replicates (= independent cultures in different wells of 96 well-plate, or independent culture tubes) were measured for the determination of P. denitrificans growth rates.<br>Four biological replicates (= independent cultures) per condition were used to generate biomass for proteomic analysis.
Data exclusions	No data were excluded from the analyses.
Replication	Controls verifying same levels of specific activities between different enzyme preparations were performed routinely. When applicable, we performed our experiments using multiple independent samples (e.g., independent cultures). All attempts at replication of our findings were successful.
Randomization	The microorganisms used in this study were selected and divided randomly in the different conditions. No criteria of selection were applied.

Blinding	Blinding of samples was not applicable for the kind of experiments included in this study. Blinding was not possible because the person in charge of the analysis was the one responsible for taking the samples.

Reporting for specific materials, systems and methods

We require information from authors about some types of materials, experimental systems and methods used in many studies. Here, indicate whether each material, system or method listed is relevant to your study. If you are not sure if a list item applies to your research, read the appropriate section before selecting a response.

Materials & experimental systems

n/a	Involved in the study	
☒	☐	Antibodies
☒	☐	Eukaryotic cell lines
☒	☐	Palaeontology and archaeology
☒	☐	Animals and other organisms
☒	☐	Clinical data
☒	☐	Dual use research of concern
☒	☐	Plants

Methods

n/a	Involved in the study	
☒	☐	ChIP-seq
☒	☐	Flow cytometry
☒	☐	MRI-based neuroimaging

Plants

Seed stocks	No research involving plants is reported in this manuscript.
Novel plant genotypes	No research involving plants is reported in this manuscript.
Authentication	No research involving plants is reported in this manuscript.
